# Laparoscopic Common Bile Duct Exploration for Choledocholithiasis in a Patient with Situs Inversus Totalis and Prior Total Gastrectomy with Roux-en-Y Reconstruction: A Case Report

**DOI:** 10.70352/scrj.cr.25-0608

**Published:** 2025-11-11

**Authors:** Tomoyuki Nagata, Yuya Arimura, Masahiro Kojo, Kenichi Takemoto, Kei Naito, Katsunori Nakano

**Affiliations:** Department of Surgery, Omihachiman Community Medical Center, Omihachiman, Shiga, Japan

**Keywords:** situs inversus totalis, laparoscopic common bile duct exploration, Roux-en-Y reconstruction, choledocholithiasis, case report

## Abstract

**INTRODUCTION:**

Situs inversus totalis (SIT) is a rare congenital condition characterized by mirror-image transposition of the thoracic and abdominal viscera. Herein, we report a rare case of laparoscopic common bile duct exploration with choledochotomy and stone extraction in a patient with SIT and a history of total gastrectomy with Roux-en-Y (R-Y) reconstruction.

**CASE PRESENTATION:**

A 70-year-old woman with SIT and a history of total gastrectomy with R-Y reconstruction presented with recurrent cholangitis caused by choledocholithiasis. Conservative therapy led to temporary improvement, but symptoms recurred after 2 months. Percutaneous transhepatic gallbladder drainage was followed by laparoscopic cholecystectomy and laparoscopic common bile duct exploration (LCBDE) one week later. Dense adhesions along the midline and right upper quadrant prevented standard port placement, so all maneuvers were confined to the left abdomen. A 3-cm umbilical minilaparotomy accommodated a Lap Protector with two 5-mm working ports, and additional 5-mm ports were inserted in the left lower abdomen for the camera, in the left upper abdomen for the assistant, and above the choledochotomy site for choledochoscope access. CT confirmed complete SIT and an 18-mm common bile duct (CBD) stone with upstream dilation. Endoscopic retrograde cholangiopancreatography was impossible because the long Roux limb and adhesions limited endoscopic access. Choledochotomy was aided by traction sutures; the impacted stone was fragmented intraductally and retrieved with basket forceps. The CBD was closed primarily without drainage. The postoperative course was uneventful, and the patient was discharged on day 8. No complications occurred during the 4-week follow-up.

**CONCLUSIONS:**

This case emphasizes the technical challenges of laparoscopic biliary surgery in patients with situs inversus and a history of R-Y reconstruction. The key points include individualized port placement, surgeon positioning, traction, and secure primary closure. LCBDE is feasible in patients with SIT and prior R-Y reconstruction if surgical strategies are individualized based on anatomical variation and surgical history.

## Abbreviations

CBD
: common bile duct
ERCP
: endoscopic retrograde cholangiopancreatography
LC
: laparoscopic cholecystectomy
R-Y
: Roux-en-Y
SIT
: situs inversus totalis

## INTRODUCTION

Situs inversus totalis (SIT) is a rare congenital condition characterized by complete mirror-image transposition of the thoracic and abdominal viscera, with an incidence of approximately 1 in 10000–20000 individuals.^1,2)^ SIT itself does not predispose patients to biliary disease; however, it poses significant challenges for both diagnosis and surgical management due to an altered anatomical orientation.^2,3)^

Cholelithiasis and choledocholithiasis are conditions commonly observed in the general population. Nevertheless, their management in patients with SIT requires cautious adjustment of surgical techniques, including port placement, surgeon positioning, and dissection strategies. Previous reports have shown that laparoscopic cholecystectomy is feasible in patients with SIT,^1,3–7)^ and a few cases of laparoscopic common bile duct exploration (LCBDE) have also been reported.^8–10)^

In addition, an altered anatomy after total gastrectomy with Roux-en-Y (R-Y) reconstruction further complicates the endoscopic management of choledocholithiasis. Endoscopic retrograde cholangiopancreatography (ERCP) is often technically impossible in such patients. Thus, surgical intervention is the preferred option.^11–13)^ Recent reports have further expanded the evidence base regarding laparoscopic bile duct surgery in patients with situs inversus. For instance, Matsuura et al. described LCBDE using a 3D imaging system,^14)^ while Krishna et al. reported a technically demanding case of laparoscopic bile duct exploration in a patient with situs inversus and altered anatomy.^15)^ These studies highlight that despite its feasibility, the procedure requires careful adaptation to individual anatomical variations. To the best of our knowledge, reports describing LCBDE in a patient with a combined history of SIT and total gastrectomy with R-Y reconstruction remain extremely limited.

Herein, we report a rare case of laparoscopic choledocholithotomy and stone extraction for choledocholithiasis in a 70-year-old woman with SIT and a history of total gastrectomy with R-Y reconstruction, highlighting the technical challenges and key surgical strategies.

## CASE PRESENTATION

**Fig. 1:**
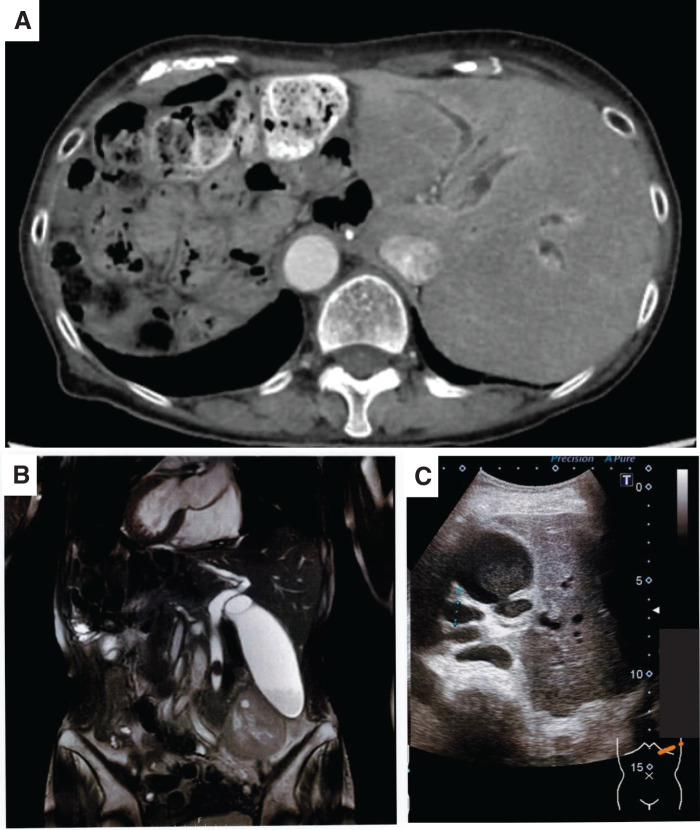
Preoperative imaging findings. (**A**) Contrast-enhanced CT showing mirror-image positioning of abdominal organs consistent with situs inversus totalis. (**B**) Magnetic resonance cholangiopancreatography revealing an 18-mm stone within the common bile duct, accompanied by upstream biliary dilation. (**C**) Ultrasonography demonstrating choledocholithiasis with posterior acoustic shadowing.

A 70-year-old female with a known history of SIT and prior total gastrectomy with R-Y reconstruction presented with fever and left upper quadrant abdominal pain. Laboratory tests revealed elevated inflammatory markers and cholestatic liver enzymes. Imaging confirmed choledocholithiasis with upstream biliary dilation in a mirror-image anatomical setting (**Fig. 1**). Initial management included broad-spectrum antibiotics and biliary decompression. ERCP was attempted by the gastroenterology team; however, it failed because the long Roux limb and dense postoperative adhesions prevented the endoscope from reaching the papilla. Therefore, the patient was referred to the surgical department. After the patient’s informed consent was obtained, we planned a 2-stage approach consisting of laparoscopic cholecystectomy followed by LCBDE.

### Operative findings and technique

**Fig. 2:**
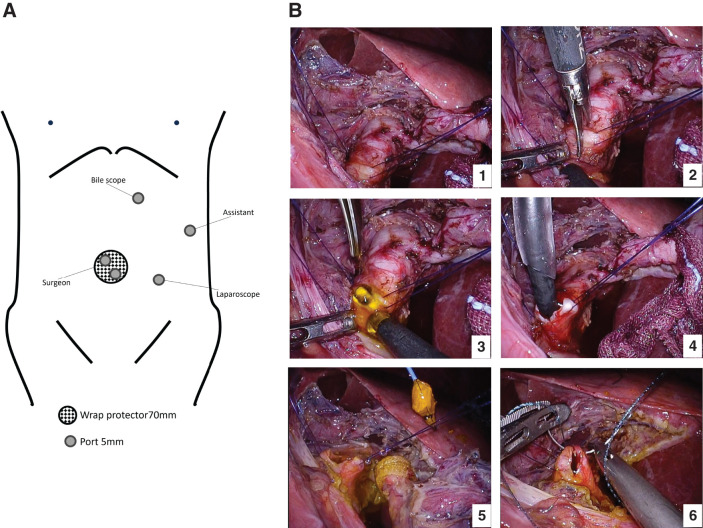
Intraoperative findings. (**A**) Port placement schema showing the modified arrangement under severe adhesions. A 3-cm mini-laparotomy was created at the umbilicus, through which a Lap Protector was inserted. This incision was also used to insert two 5-mm working ports for the operator’s instruments. A 5-mm camera port was positioned in the left lower abdomen, while a 5-mm assistant port was inserted in the left upper abdomen. An additional 5-mm port was placed just above the planned choledochotomy site for direct choledochoscope access. This arrangement enabled all operative maneuvers to be conducted entirely within the left abdominal cavity. (**B**) Choledochotomy with traction sutures placed on the common bile duct to facilitate exposure. Impacted stones were fragmented intraductally and extracted using basket forceps. Closure was completed with interrupted sutures at both ends and a continuous running barbed suture in between.

Dense adhesions were encountered along the midline, precluding standard port placement (**Fig. 2A**). Therefore, we adopted a left-sided port strategy, confining all maneuvers to the left abdominal quadrants. After the hepatoduodenal ligament was exposed through adhesiolysis, traction sutures were placed on the common bile duct (CBD) to improve exposure, and a longitudinal choledochotomy was performed. The impacted 18-mm stone was partially fragmented intraductally for size reduction and then retrieved using a basket forceps (FG-55D; Olympus Corporation, Tokyo, Japan) under direct vision with a 3.4-mm flexible choledochoscope (CHF-V2; Olympus) (**Fig. 2B**). A 5-mm flexible laparoscope (VISERA ELITE III OTV-S700; Olympus) and 5-mm trocars (E·Z Trocar Smart Insertion SG953-V12; Hakko, Nagano, Japan) were used in combination with a Lap Protector mini type (Hx0707; Hakko) and an EZ Access port (FF07; Hakko). Stay sutures for traction on the CBD were placed using 3-0 VICRYL Plus antibacterial absorbable sutures (VCP311H; Ethicon, Somerville, NJ, USA). Choledochotomy was then completely closed using interrupted sutures at both ends and a continuous 4-0 V-Loc 180 absorbable barbed suture (V-20 taper needle; VLOCL0603; Medtronic (Covidien), Mansfield, MA, USA) in between. For adhesiolysis and tissue dissection, Harmonic ACE+7 ultrasonic shears (Ethicon) were used. After stone extraction, the bile duct was re-explored with the choledochoscope to ensure that no fragments remained. Intraoperative cholangiography was omitted because direct visualization through the choledochoscope provided adequate assessment, and the cystic duct was unavailable for cannulation in accordance with the surgical plan. The bile flow was smooth, with no evidence of intrahepatic ductal hypertension; thus, no C-tube or external biliary drainage was placed. This strategy aligns with contemporary evidence showing that post-LCBDE primary duct closure (PDC) is safe and effective compared with T-tube drainage and can be considered a first-line treatment option.^16,17)^ Representative selection criteria for PDC include a CBD diameter ≥8 mm, complete duct clearance, and no distal strictures or malignancy. When these conditions are met, PDC without intraluminal drainage is feasible and safe.^18–20)^ In the present case, the CBD was dilated (18 mm on preoperative imaging), choledochoscopy confirmed complete duct clearance, and no distal obstruction was identified, thereby meeting the criteria.

### Postoperative course

**Fig. 3:**
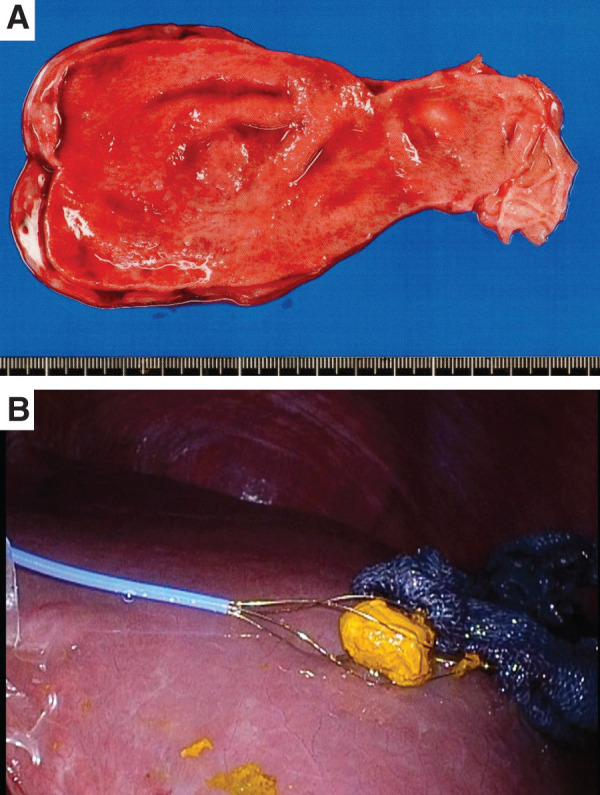
Resected specimens. (**A**) Gallbladder and (**B**) common bile duct stone measuring 18 mm in diameter.

The postoperative course was uneventful. Oral intake resumed on POD 2, and the patient was discharged on POD 8. During the 4-week follow-up, no complications were observed, and liver function tests normalized. The resected gallbladder and extracted CBD stone are shown in **Fig. 3**.

## DISCUSSION

SIT is a rare congenital anomaly, with an incidence of approximately 1 in 10000–20000 individuals.^1,2)^ The occurrence of choledocholithiasis itself is not uncommon after gastrectomy. However, patients who undergo R-Y reconstruction present unique diagnostic and therapeutic challenges because ERCP is technically difficult or often impossible to perform.^11–13)^ In our case, ERCP was unsuccessful because the endoscope could not reach the papilla, given the long Roux limb and dense postoperative adhesions. Although reported as a possible alternative, balloon-assisted ERCP was considered impractical in this situation because of the extensive adhesions and complex anatomy. Therefore, surgical intervention remains the mainstay of treatment in such cases, particularly in moderate acute cholangitis, as defined by the Tokyo Guidelines 2018, in which timely biliary decompression and definitive stone removal are required. The combination of SIT, a history of total gastrectomy with R-Y reconstruction, and LCBDE makes the current case remarkably rare and clinically significant.

From a diagnostic perspective, situs inversus can delay the identification of biliary disease because of atypical symptom localization.^2,3)^ Patients frequently exhibit left-sided upper abdominal pain rather than the typical right-sided pain seen in orthotopic anatomy. In our case, recurrent cholangitis was ultimately confirmed via imaging, which showed choledocholithiasis in the mirror-image anatomy.

Technical challenges are another important consideration. In patients with situs inversus, port placement and surgeon positioning must be individualized based on the mirror-image anatomy. However, a strict mirror-image configuration is not always optimal. Rather than simply mirroring the anatomy, the surgeon’s dominant hand and ergonomic factors should also be considered in port placement. Previous reports of SIT cholecystectomy have emphasized similar adjustments.^3,4)^ In our case, the surgery was performed via left-sided ports due to dense adhesions, further emphasizing the need for individualized strategies beyond mirror-image placement.^3–5)^ Furthermore, because of dense adhesions around the midline, the surgery was completed using left-sided ports only, thereby confining all operative maneuvers strictly to the left half of the abdominal cavity, a strategy that allowed safe access despite the altered anatomy.

In addition, based on the operative experience, several technical tips should be highlighted. First, placement of traction sutures on the CBD provided effective exposure and facilitated choledochotomy, a maneuver also described in previous reports.^8,9)^ Second, impacted stones were fragmented within the duct, size-reduced to improve mobility, and then retrieved with basket forceps. This underscores the importance of preparing multiple retrieval devices.^10)^ Third, primary closure with a barbed suture enabled secure, efficient repair without requiring T-tube drainage, aligning with contemporary evidence favoring PDC^14,15)^ and reports supporting barbed suture use for choledochotomy closure.^21–23)^

Comparison with the existing literature reveals both similarities and differences. Some reports have shown that laparoscopic cholecystectomy is feasible in patients with situs inversus, confirming its safety when performed by experienced professionals.^1–7)^ The number of studies on LCBDE in situs inversus is significantly limited,^8–10)^ though newer case reports have been published.^14,15)^ In general, after total gastrectomy with R-Y reconstruction, retrograde endoscopic access to the CBD is technically challenging.^11–13)^ The current case is unique as it shows the coexistence of complete situs inversus and prior total gastrectomy with R-Y reconstruction, as well as the successful completion of LCBDE using a left-sided port strategy under dense adhesions. Importantly, the fact that the procedure could be completed laparoscopically provided the additional benefit of accomplishing definitive treatment in a minimally invasive manner, thereby underscoring the value of laparoscopic management as an alternative to open surgery.^24)^

The novelty of this report lies in three major aspects. First, the coexistence of SIT and prior total gastrectomy with R-Y reconstruction is exceptionally rare, presenting a unique combination of reversed anatomy and surgically altered biliary access. Second, the procedure was successfully completed using an entirely left-sided port approach, which differs from the commonly reported mirror-image configuration and flexibly adapts to severe adhesions. Third, the technical combination of intraductal stone fragmentation and primary closure with a barbed suture safely and effectively managed the ducts without utilizing C- or T-tube drainage. Together, these features highlight an individualized, minimally invasive strategy that expands the technical options for complex biliary surgery in patients with altered anatomy.

Finally, the postoperative course in our case was uneventful, which is consistent with previous reports showing that situs inversus itself does not increase the risk of postoperative complications.^1,2)^ Rather, the success of such procedures is significantly dependent on cautious preoperative planning, adaptation of surgical strategies, and the use of meticulous intraoperative techniques.

This case shows that LCBDE is feasible and safe in patients with SIT, even in the complex setting of previous total gastrectomy with R-Y reconstruction, provided that appropriate technical modifications are applied.

## CONCLUSIONS

LCBDE can be safely and effectively performed even in patients with SIT and prior total gastrectomy with R-Y reconstruction. Cautious preoperative planning, individualized adjustment of port placement and surgeon positioning, and technical refinements, such as the use of traction sutures and secure PDC, are essential for achieving a successful outcome. Based on our experience, laparoscopic management is a feasible and valuable option when endoscopic intervention is not possible due to a complex anatomy.

## DECLARATIONS

### Availability of data and materials

The dataset(s) supporting the conclusions of this article are included within the article. Additional information is available from the corresponding author upon reasonable request.

## References

[1] Salama IA, Abdullah MH, Houseni M. Laparoscopic cholecystectomy in situs inversus totalis: feasibility and review of literature. Int J Surg Case Rep 2013; 4: 711–5. 23810920 10.1016/j.ijscr.2013.02.030 PMC3710908

[2] Enciu O, Toma EA, Tulin A, et al. Look beyond the mirror: laparoscopic cholecystectomy in situs inversus totalis — a systematic review and meta-analysis (and report of new technique). Diagnostics (Basel) 2022; 12: 1265. 35626419 10.3390/diagnostics12051265 PMC9140146

[3] He T, Zou J. Laparoscopic cholecystectomy in a patient with situs inversus totalis presenting with cholelithiasis: a case report. Front Surg 2022; 9: 874494. 35495755 10.3389/fsurg.2022.874494 PMC9046872

[4] Du T, Hawasli A, Summe K, et al. Laparoscopic cholecystectomy in a patient with situs inversus totalis: port placement and dissection techniques. Am J Case Rep 2020; 21: e924896. 32886654 10.12659/AJCR.924896 PMC7491955

[5] Matsumoto T, Okawa T, Asakura Y, et al. Laparoscopic cholecystectomy in a patient with situs inversus totalis: report of a case with surgical tips. AME Surg J 2023; 3: 10.

[6] Lakhey P, Shrestha M, Sharma R, et al. Laparoscopic cholecystectomy in situs inversus totalis: a case report. Clin Case Rep 2025; 13: e70222. 39967839 10.1002/ccr3.70222 PMC11833166

[7] Abu-Oddos N, Abu-Jeyyab M, Al Mse’adeen M, et al. Laparoscopic cholecystectomy in a patient with situs inversus totalis and a double superior vena cava. Am J Case Rep 2023; 24: e938774. 37099479 10.12659/AJCR.938774 PMC10152506

[8] Kang SB, Han HS. Laparoscopic exploration of the common bile duct in a patient with situs inversus totalis. J Laparoendosc Adv Surg Tech A 2004; 14: 103–6. 15107220 10.1089/109264204322973880

[9] Fukami Y, Hasegawa H, Sakamoto E, et al. Laparoscopic choledocholithotomy in situs inversus totalis: a case report. Tando 2006; 20: 188–92. (in Japanese)

[10] Wong J, Tang CN, Chau CH, et al. Laparoscopic cholecystectomy and exploration of the common bile duct in a patient with situs inversus. Surg Endosc 2001; 15: 218. 10.1007/s004640040037 12200663

[11] Khara HS, Parvataneni S, Park S, et al. Review of ERCP techniques in Roux-en-Y gastric bypass patients: highlight on the novel EUS-directed transgastric ERCP (EDGE) technique. Curr Gastroenterol Rep 2021; 23: 10. 34212281 10.1007/s11894-021-00808-3 PMC8249251

[12] Samarasena JB, Nguyen NT, Lee JG. Endoscopic retrograde cholangiopancreatography in patients with Roux-en-Y anatomy. J Interv Gastroenterol 2012; 2: 78–83. 23687591 10.4161/jig.22203 PMC3655346

[13] Yachimski P, Guggilapu S, Poulose B. Laparoscopic-assisted transgastric endoscopic retrograde cholangiopancreatography: a review of indications, technical considerations, and outcomes. Ann Laparosc Endosc Surg 2024; 9: 26.

[14] Matsuura H, Haruta H, Suzuki T, et al. Laparoscopic cholecystectomy and laparoscopic common bile duct exploration for cholecystolithiasis and choledocholithiasis in a patient with situs inversus totalis: a case report. Asian J Endosc Surg 2024; 17: e13346. 38943368 10.1111/ases.13346

[15] Krishna S, Raja K, Pottakkat B. Laparoscopic cholecystectomy with common bile duct exploration for choledocholithiasis in a patient with situs inversus totalis – case report and review of literature. Int J Surg Case Rep 2025; 130: 111238. 40203625 10.1016/j.ijscr.2025.111238 PMC12005859

[16] Podda M, Polignano FM, Luhmann A, et al. Systematic review with meta-analysis of studies comparing primary duct closure and T-tube drainage after laparoscopic common bile duct exploration for choledocholithiasis. Surg Endosc 2016; 30: 845–61. 26092024 10.1007/s00464-015-4303-x

[17] Zhu T, Lin H, Sun J, et al. Primary duct closure versus T-tube drainage after transductal laparoscopic common bile duct exploration: an updated meta-analysis. J Zhejiang Univ Sci B 2021; 22: 985–1001. 34904412 10.1631/jzus.B2100523 PMC8669324

[18] Haggerty S, et al. Clinical spotlight review: laparoscopic common bile duct exploration. Society of American Gastrointestinal and Endoscopic Surgeons (SAGES). Available at: https://www.sages.org/publications/guidelines/clinical-spotlight-review-laparoscopic-common-bile-duct-exploration/ (accessed 15 Oct 2025).

[19] Lai W, Xu N. Feasibility and safety of choledochotomy primary closure in laparoscopic common bile duct exploration without biliary drainage: a retrospective study. Sci Rep 2023; 13: 22473. 38110402 10.1038/s41598-023-49173-3 PMC10728103

[20] Shen D, Chang P, Xu H, et al. Is an 8 mm cutoff necessary when performing primary common bile duct closure after laparoscopic common bile duct exploration? Pak J Med Sci 2024; 40: 2636–42. 39634899 10.12669/pjms.40.11.9441 PMC11613372

[21] Zhou H, Wang S, Fan F, et al. Primary closure with knotless barbed suture versus traditional T-tube drainage after laparoscopic common bile duct exploration: a single-center medium-term experience. J Int Med Res 2020; 77: 108–13. 10.1177/0300060519878087 PMC7262853 31612768

[22] Zhu J, Zhang Y, Gong J, et al. Closure of choledochotomy with a barbed absorbable suture after laparoscopic common bile duct exploration. Am Surg 2023; 33: 56–60. 10.1177/0003134820982861 33351645

[23] Tan YP, Lim C, Junnarkar SP, et al. 3D laparoscopic common bile duct exploration and primary repair with absorbable barbed suture is safe and feasible. J Clin Transl Res 2021; 7: 473–8. 34667894 PMC8520704

[24] De Silva HM, Howard T, Bird D, et al. Outcomes following common bile duct exploration versus endoscopic stone extraction before, during and after laparoscopic cholecystectomy for patients with common bile duct stones. HPB (Oxford) 2022; 24: 2125–33. 36130852 10.1016/j.hpb.2022.08.014

